# Functional interpretation of biophysical properties of spiking neurons

**DOI:** 10.1186/1471-2202-14-S1-P104

**Published:** 2013-07-08

**Authors:** Fleur Zeldenrust, Boris S Gutkin, Sophie Denève

**Affiliations:** 1 Group for Neural Theory, École Normale Supérieure, Département d'Études Cognitives, 29, rue d'Ulm,75005 Paris, France

## 

Neurons in the sensory systems extract information about the outside world from a constant stream of noisy sensory inputs. Previously, we have shown that the dynamics of spiking sensory neurons can be interpreted as a form of Bayesian inference in time [1], where spikes are only fired if they provide new information that cannot be predicted from past activity. Bayesian integration results in two characteristic nonlinearities: spike-frequency adaptation and rectification of negative (hyperpolarizing) inputs. We find that these should be inherently coupled. For example, the time constant of integration of the neuron affects both the time constant of the rectification (a property of synaptic integration) as well as the spike-frequency adaptation (the output mechanism of the neuron). Therefore, we predict that in order to perform optimal inference, these two mechanisms should systematically co-vary and be coupled to the characteristics of the next processing layer.

In order to compare the nonlinearities of Bayesian integration to biophysical properties, an explicit quantitative link between the abstract Bayesian neuron and specific biophysical models has to be made. For example, one central issue is how to rescale the input. We show that this can be done using the rheobase of each model and the linear part of the input-frequency curve. Following this rescaling, the parameters of the Bayesian neuron can be fitted to specific biophysical models or experiments using the model fitting toolbox [2,3] in Brian [4].

We find that neurons can function optimally in two different biologically relevant input regimes: a sparse coincidence detection regime where most of the synapses are excitatory, firing rates are low and EPSCs are strong (similar to Softky and Koch [5,6]); and a balanced regime, where excitation and inhibition are both strong and approximately balanced and output firing rates are much higher (similar to Shadlen and Newsome [7,8]). In all other regimes, neurons cannot reliably detect the stimulus from their inputs. We determine the range of biophysical parameters that insure optimal stimulus detection and provide a quantitative link between the dynamics inherent in the biophysics of single neurons and their function, i.e. the statistical properties of the stimulus they optimally detect.
